# Altered expression of stromal interaction molecule (STIM)-calcium release-activated calcium channel protein (ORAI) and inositol 1,4,5-trisphosphate receptors (IP_3_Rs) in cancer: will they become a new battlefield for oncotherapy?

**DOI:** 10.1186/s40880-016-0094-2

**Published:** 2016-03-24

**Authors:** Jing Wen, Ying-Cheng Huang, Huan-Huan Xiu, Zhi-Ming Shan, Kang-Qing Xu

**Affiliations:** Department of Anesthesiology, The First Affiliated Hospital, Sun Yat-sen University, Guangzhou, Guangdong 510080 P. R. China; Zhongshan School of Medicine, Sun Yat-sen University, Guangzhou, Guangdong 510080 P. R. China

**Keywords:** Stromal interaction molecule (STIM), Calcium release-activated calcium channel protein (ORAI), Inositol 1,4,5-trisphosphate receptors (IP_3_Rs), Ca^2+^, Tumorigenesis

## Abstract

The stromal interaction molecule (STIM)-calcium release-activated calcium channel protein (ORAI) and inositol 1,4,5-trisphosphate receptors (IP_3_Rs) play pivotal roles in the modulation of Ca^2+^-regulated pathways from gene transcription to cell apoptosis by driving calcium-dependent signaling processes. Increasing evidence has implicated the dysregulation of STIM–ORAI and IP_3_Rs in tumorigenesis and tumor progression. By controlling the activities, structure, and/or expression levels of these Ca^2+^-transporting proteins, malignant cancer cells can hijack them to drive essential biological functions for tumor development. However, the molecular mechanisms underlying the participation of STIM–ORAI and IP_3_Rs in the biological behavior of cancer remain elusive. In this review, we summarize recent advances regarding STIM–ORAI and IP_3_Rs and discuss how they promote cell proliferation, apoptosis evasion, and cell migration through temporal and spatial rearrangements in certain types of malignant cells. An understanding of the essential roles of STIM–ORAI and IP_3_Rs may provide new pharmacologic targets that achieve a better therapeutic effect by inhibiting their actions in key intracellular signaling pathways.

## Background

Calcium signals are widespread and rigorously regulate the majority of fundamentally important physiologic processes ranging from cell proliferation to cell apoptosis [1]. The precise and tightly controlled intracellular calcium ion concentration depends on finely tuned modulation by various calcium-transporting processes, including Ca^2+^ channels, pumps, and receptors [2]. These Ca^2+^-transporting molecules strictly regulate the transient or sustained waves, spikes, or oscillations of Ca^2+^ signaling in different cellular compartments and microdomains to maintain a delicate balance between feeding into the cytoplasm and releasing from internal Ca^2+^ stores [3]. Any perturbation and disorder of the delicate Ca^2+^ homeostasis may lead to long-ranging consequences. Therefore, it is not surprising that any derangement of Ca^2+^ channels and/or receptors will contribute to the establishment of many life-threatening diseases, such as cardiopathy [4], heart failure [5], neurodegenerative diseases [6], and cancer [7]. Remarkably, the altered expression or activity of these Ca^2+^ channels or receptors is characterized by the features of specific cancer subtypes, of which the most important is the protein complex consisting of the stromal interaction molecule (STIM), calcium release-activated calcium channel protein (ORAI), and inositol 1,4,5-trisphosphate (IP_3_) receptors (IP_3_Rs). STIM–ORAI is able to sense and respond to intracellular Ca^2+^ microenvironmental changes that occur during cancer development. This complex primarily mediates the Ca^2+^ influx, with STIM serving as the endoplasmic reticulum (ER) Ca^2+^ sensor and ORAI as the Ca^2+^-selective entry channel. Furthermore, the roles of IP_3_Rs are also discussed. There is emerging evidence that IP_3_Rs, which regulate the Ca^2+^ flux from the ER into the cytosol and mitochondria, play crucial roles in the apoptotic pathway; additionally, IP_3_Rs have been implicated in cellular senescence [3]. The last decade in clinical oncology has been noteworthy because of advances in our understanding of the derangement of Ca^2+^ channels/transporters that are thought to be responsible for the development of cancers [8]. Although enormous explorations have been performed, the molecular mechanisms by which these derangements affect tumorigenesis and tumor progression are far from being fully understood. In this review, we discuss recent progress in understanding the roles of STIM–ORAI and IP_3_Rs, with a focus on exploring the mechanism underlying the hijacking of the Ca^2+^-transporting molecules STIM-ORAI and IP_3_Rs by malignant cancer cells that leads to tumor onset, growth, and metastasis. The concomitant interaction between STIM–ORAI and IP_3_Rs is also discussed. Understanding the molecular basis and pathologic transformations of Ca^2+^-transporting molecules in cancer cells will offer an opportunity for pharmacologic modulation and therapeutic intervention.

## The structure and function of the STIM and ORAI protein families

External stimulation leads to an increase in cytoplasmic Ca^2+^ from either the entry of extracellular Ca^2+^ across the plasma membrane or the release of Ca^2+^ from internal calcium stores in the ER and sarcoplasmic reticulum (SR) [9]. Both of these functions involve the permeable Ca^2+^ channels that are located on the plasma membrane. Upon the stimulation, extracellular Ca^2+^ can enter the cytoplasm through Ca^2+^ channel transport. Moreover, the plasma membrane is responsible for refilling the internal Ca^2+^ stores when they are depleted. The primary Ca^2+^ entry pathway is store-operated Ca^2+^ entry (SOCE), which includes two key components: a sensitive sensor of calcium store depletion (STIM) and an effective channel that can facilitate calcium entry into the cell (ORAI) [10–12].

STIM, which is predominantly located in the ER, was identified using an RNA interference screen in Drosophila S2 cells; then, two mammalian orthologs (STIM1 and STIM2) were found [13]. Both STIM1 and STIM2 act as sensors of Ca^2+^ store levels in the ER and control calcium refilling by forming connections with ORAI [14]. STIM1 contains an ER luminal N-terminus and a cytosolic C-terminus. The ER luminal portion consists of a canonical Ca^2+^-binding EF-hand (a conventional helix-loop-helix EF motif), a hidden EF hand, and a sterile α-motif (SAM) domain. The cytosolic strand includes three putative coiled-coil (CC1, CC2, and CC3) regions, calcium release-activated calcium (CRAC) modulatory domain (CAD) or a STIM-ORAI-activating region (SOAR), serine or proline-rich segments, and lysine-rich regions [15]. The low Ca^2+^-binding affinity of EF-SAM perfectly matches the detailed alteration of the Ca^2+^ concentration and enables the ER sensor protein to respond to changes in the Ca^2+^ concentration in ER. The Ca^2+^ depletion in the ER leads to the dissociation of Ca^2+^ from the EF hand, thereby destabilizing the entire EF-SAM entity. The CC regions and the serine/proline-rich region promote the oligomerization of STIM, thereby enabling its redistribution into multiple punctae and its localization at ER-plasma membrane junctions. The CC domain has been proven to control the exposure and oligomerization of the SOAR [16]. The structure of STIM2 is similar to that of STIM1; however, STIM1 is widely expressed at both the cell surface and the ER, whereas STIM2 is expressed only in the intracellular space. A growing number of studies have indicated that STIM2 is a potent inhibitor and a feedback regulator of STIM1 via preventing it from forming an aggregate to stabilize basal concentrations of Ca^2+^ in the cytosol and ER [17]. Thus, STIM2 is regarded as a critical regulator of basal Ca^2+^ levels in the human signaling proteome, whereas STIM1 seems to be more involved in the Ca^2+^ entry associated with more pronounced depletion [18, 19].

The ORAI gene encodes a family consisting of three proteins (ORAI1, ORAI2, and ORAI3). Each of these proteins consists of 4 TM-spanning segments and 3 cytosolic strands, which include the N-terminus, the second loop con-necting TM2 and TM3, and the C-terminus [20]. The ORAI1 C-terminus forms the cytosolic extension, and the C-terminal putative CC domain is important for binding to the SOAR/CAD domain of STIM1. By binding to the intracellular C-terminus, STIM1 recruits ORAI to the puncta in the plasma membrane.

**Fig. 1:**
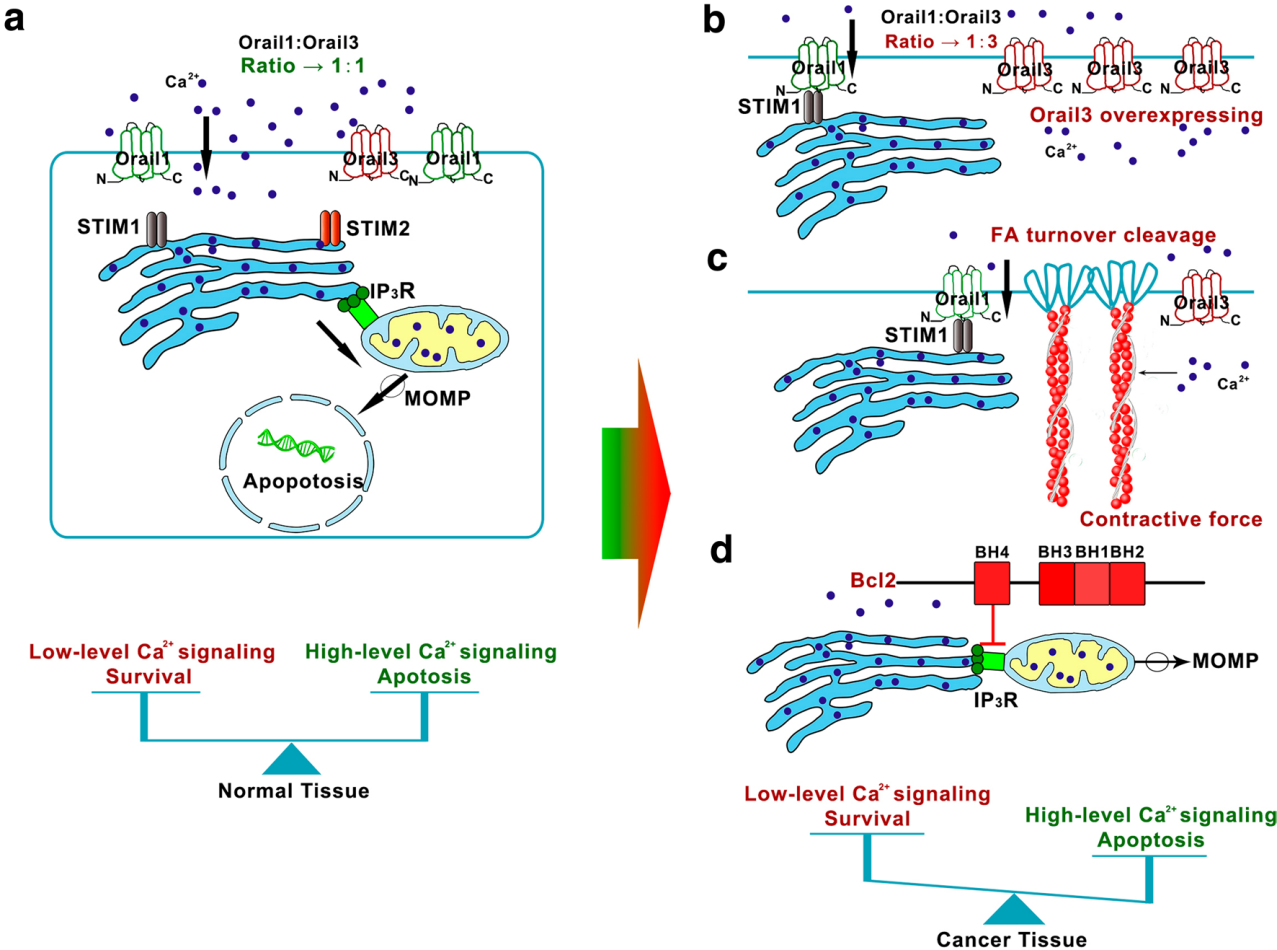
Disrupted dynamic equilibrium of stromal interaction molecule 1 (STIM1)-calcium release-activated calcium channel protein 1 (ORAI1) and inositol 1,4,5-trisphosphate receptor (IP_3_R)-mediated Ca^2+^ signaling in tumor biology. **a** In normal cells, STIM1 exists as a single-transmembrane protein in the endoplasmic reticulum (ER). The STIM1 canonical Ca^2+^-binding EF-hand (a conventional helix-loop-helix EF motif) can sensitively detect the depletion of ER luminal Ca^2+^, leading to STIM1 oligomerization and interactions with the C-terminus of ORAI1. The STIM1–ORAI1 complex controls the opening of the Store-operated Ca^2+^ entry (SOCE) channel ORAI1, thereby allowing Ca^2+^ entry. The increased Ca^2+^ in the ER can enter into the mitochondria via IP_3_Rs, leading to mitochondrial Ca^2+^ overload and indirectly causing apoptosis. The mitochondrial outer membrane permeabilization (MOMP) is considered a critical step during the point-of-no-return apoptosis in the mitochondria. **b** In prostate cancer cells, an increase in the level of the endogenous ORAI3 protein causes the association of ORAI3 with ORAI1 to form a heteromultimeric channel that can alter the ORAI3-ORAI1 ratio. These functions represent an oncogenic switch that promotes prostate cancer cell proliferation and confers apoptosis resistance. **c** STIM1–ORAI1-mediated Ca^2+^ signaling accelerates tumor cell migration through controlling focal adhesion (FA) turnover and actomyosin contractility. The STIM1–ORAI1-mediated Ca^2+^ influx regulates actomyosin formation and increases its contractile force. STIM1–ORAI1 induces the Ca^2+^ influx and promotes the cleavage of FA proteins. The *red* represents all of the factors involved in resistance to apoptosis, and the *blue* represents all of the factors that promote apoptosis. **d** Bcl-2 is a representative anti-apoptotic protein that interacts with IP_3_R via its N-terminal BH4 domain. Then, Bcl-2 inhibits the Ca^2+^ flux into the mitochondria, leading to mitochondrial Ca^2+^ deficiency and preventing cancer cell apoptosis. The deficient Ca^2+^ can break MOMP and finally prevent cancer cell apoptosis

The primary intracellular hysiologic functions of STIM and ORAI are straightforward (Fig. 1a). STIM detects the decrease in Ca^2+^ stores in the ER and moves within the ER to ER-plasma membrane junctions. Then, STIM recruits ORAI to the ER-plasma membrane junctions, where the two proteins form a close contact. The formation of the active STIM–ORAI complex at the conformational gate of the SOCE channel allows Ca^2+^ entry [21].

## The structure and function of the IP_3_ receptor family

The IP_3_R is the most ubiquitous intracellular Ca^2+^ channel, and its isoforms (IP_3_R1, IP_3_R2, and IP_3_R3) have been identified in vertebrates [22]. The majority of cell types express more than one isoform but have a predominant one. The three IP_3_R isoforms have distinct but overlapping expression patterns. IP_3_R1 is expressed in neuronal cells, IP_3_R2 is expressed in liver and muscle cells, and IP_3_R3 is expressed in most cultured cell types [23]. The general domain structure of IP_3_Rs (which exist as tetramers) has been determined; IP_3_Rs contain a binding site for IP_3_ in the N-terminal region, the channel domain, and the determinants for tetramer formation in the C-terminus [24, 25].

IP_3_Rs predominantly reside in the ER. IP_3_ is produced by phospholipase C and binds to IP_3_Rs to induce calcium release from the ER upon cell activation by endogenous or exogenous hormones, growth factors, or neurotransmitters. Notably, IP_3_-induced Ca^2+^ release is typically regulated by the Ca^2+^ concentration in the cytosol and ER. Furthermore, the activities of IP_3_Rs are biphasically regulated by cytosolic Ca^2+^. The concentration–response relationship is a typical bell-shaped curve, indicating that the IP_3_-mediated Ca^2+^ release is potentiated at a low Ca^2+^ concentration and inhibited at a higher concentration [26]. The Ca^2+^ storage in the lumen of the ER also regulates IP_3_Rs, which can prevent excessive ER depletion at low levels of store filling [27]. IP_3_R-mediated Ca^2+^ elevation regulates fundamental cellular functions, such as fertilization, cell cycle entry, cell division, metabolism, and transcription [28]. An important function of IP_3_Rs is to decide the cell fate by controlling the mitochondrial Ca^2+^ elevation and mitochondrial metabolism. Cell survival or apoptosis is encoded in the frequency and amplitude of Ca^2+^ oscillations mediated by IP_3_Rs and decoded by different Ca^2+^-sensitive kinases or phosphatases that in turn regulate the target proteins. When IP_3_Rs transport appropriate amounts of Ca^2+^ from the ER to the mitochondria, they catalyze the conversation of pyruvate to acetyl-coenzyme A (CoA) to produce adenosine triphosphate (ATP) and nicotinamide adenine dinucleotide 2′-phosphate (NADPH). The insufficient transport of Ca^2+^ to the mitochondria induces cellular autophagy. Conversely, when activated IP_3_Rs excessively transport Ca^2+^ from the ER to the mitochondria, the mitochondrial Ca^2+^ is overloaded, which induces a dissipation of the mitochondrial potential, the opening of the permeability transition pore, and the release of pro-apoptotic factors such as cytochrome *c* [29]; this process ultimately triggers cell apoptosis. Therefore, IP_3_Rs play pivotal roles in the apoptotic process via controlling the cellular response to apoptotic signals and conferring oncogenic features to the cell [30].

## The emerging roles of STIM and ORAI in tumorigenesis and tumor progression

STIM and ORAI have been found to be abundantly expressed in human cancer tissues and multiple tumor cell lines. Abnormal spatial and temporal changes in these two proteins have been found to be involved in many aspects of tumorigenesis, including cancer cell proliferation, migration, and apoptosis resistance.

### STIM and ORAI are overexpressed in tumors

Increasing evidence has shown that STIM and ORAI are overexpressed in many types of malignant tumors, including breast cancer [31], glioblastoma [32], prostate cancer [33], hepatocellular carcinoma [34], esophageal squamous cell carcinoma (ESCC) [35], and clear cell renal cell carcinoma (ccRCC) [36] (Table 1). An investigation of 24 patients with cervical cancer found that 71 % of the patients showed increased expression of STIM1 in primary cervical cancer tissues compared with non-cancerous tissues. Abnormal overexpression of STIM1 contributed to large tumor sizes and low 5-year survival rates. A similar association between STIM expression and tumor growth was also demonstrated in the study by Yang et al. [34]. The authors found that highly invasive CC-LM3 hepatocytes overexpressed STIM1 at a level approximately eightfold higher than normal LO2 hepatocytes in vitro. A study of 295 breast cancer patients obtained a similar result [37]. The survival of breast cancer patients with high *STIM1* mRNA levels in tumors was significantly reduced compared with the control group. Additionally, STIM1 could also be used as a predictive marker for metastatic potential in patients with hepatocellular carcinoma [38]. The high expression of ORAI1 also indicated a poor prognosis and depressed recurrence-free survival. In line with these findings, Zhu et al. [35] demonstrated that malignant ESCC tissues displayed an ectopic overexpression of ORAI1 compared with neighboring non-tumorous esophageal tissues. A similar result for ORAI3 in breast cancer cell lines was reported by Faouzi et al. [39], who showed that the expression of the *ORAI3* mRNA was increased in breast cancer tissues from the majority (76.9 %) of patients compared with healthy control tissues. Increased expression of ORAI3 in tumor tissues from 60 patients presenting non-small cell lung adenocarcinoma was also noted by Ay et al. [40]. Additionally, Schmidt et al. [41] showed that the expression levels of STIM and ORAI were significantly higher in cisplatin-resistant ovarian carcinoma cells than in cisplatin-sensitive cells (Table 1). These results provide evidence supporting an association between STIM–ORAI expression and poor outcomes in patients with malignant cells.

**Table 1:** Roles of the stromal interaction molecule (STIM)-calcium release-activated calcium channel protein (ORAI) and inositol 1,4,5-trisphosphate receptors (IP_3_Rs) in tumor invasion and metastasis

Channel	Cell type(s)	Mechanism and function	References
STIM-ORAI	Human breast cancer MDR-MB-231 and MCF-10A cells	Regulates focal adhesion (FA) turnover and increases tumor cell migration	[31]
	Human glioblastoma multiforme (GBM) U251-MG, SNB19, U87, and LN229 cells	Regulates GBM cell invasiveness and increases tumor metastasis	[32]
		Regulates FA turnover and epithelial-to-mesenchymal transition	[57]
	Clear cell renal cell carcinoma (RCC)	Regulates migration and proliferation; increases RCC development	[36]
	Human colorectal cancer (CRC)	Associated with tumor size, lymph node metastasis, and serum levels of carcinoembryonic antigen; promotes CRC migration; remolds Ca^2+^ signal and channel features	[48]
	Pancreatic adenocarcinoma Panc1 cells	Protects tumor against apoptosis	[49]
	Human melanoma SK-Mel-2 and SK-Mel-24 cells	Promotes melanoma cells proliferation and migration	[50]
ORAI	Human prostate epithelial cell line DU145 cells	Alters the molecular components of ORAI; increases ORAI1:ORAI3 ratio; and is associated with negative prognosis	[9]
	Human esophageal squamous cell carcinoma KYSE-150, -190, -30, -510, and -790 cells	Regulates cell proliferation, migration, and invasion; promotes tumor growth; and is associated with recurrence rate	[43]
STIM1	Hepatocellular carcinoma (HCC) HepG2, Hep3B, HCC-LM3, and Huh7 cells	Enhances FA turnover; increases HCC migration	[34]
	Non-small cell lung cancer A549 and H460 cells	Knocks down STIM1; enhances the apoptosis induced by cisplatin	[40]
IP_3_R	Estradiol-induced breast cancer MCF-7 cells	Inhibits the growth of MCF-7 cells via the IP_3_R inhibitor caffeine	[63]
	Glioblastoma cells	blocks the glioblastoma invasion and migration via inhibiting the functions of IP_3_R	[71]
	Colorectal cancer cell lines HCT116 and DLD-1 cells	IP_3_R isoform is remodeled by oncogenic k-Ras; increases resistance to apoptosis	[72]
	Colorectal carcinoma	Increases resistance to apoptosis-mediated Ca^2+^ signal between the endoplasmic reticulum and mitochondria	[78]

### STIM and ORAI: pivotal roles in cancer development

The functions of STIM and ORAI in certain types of cancer have fascinated many investigators. The use of pharmacologic interference and small interfering RNA (siRNA)-mediated gene knockdown approaches to down-regulate STIM and ORAI at both the mRNA and protein levels inhibits tumor cell proliferation, promotes cell apoptosis, and reduces tumor size. These results revealed that STIM and ORAI promoted tumorigenesis and tumor progression through the following key events: elevated proliferation, enhanced migration, and increased resistance to apoptosis.

A study of STIM1 indicated that the gene locus encoding STIM1 on chromosome 11p15 was deleted in human rhabdomyosarcoma and rhabdoid tumor cell lines [42]. Ectopic overexpression of STIM1 in vitro could induce morphologic changes in rhabdomyosarcoma cells and ultimately lead to cell death. Therefore, STIM was a suspected tumor suppressor. However, Gueguinou et al. [43] demonstrated that knockdown of STIM1 did not inhibit the migration of breast cancer cells. Moreover, Zhu et al. [35] showed that there was no significant difference between tumor tissues and normal tissues from patients with ESCC. These results implied that STIM1 might play a nonessential role in cancer metastatic processes. These contradictory findings imply that the features and expression of STIM vary in different cancer tissues and stages.

Compared with STIM, the role of ORAI in tumorigenesis may be more explicit. The dysregulation of ORAI is affected by the activation of proto-oncogenes or the inactivation of tumor suppressors. Recently, compelling evidence has suggested that ORAI3 is closely related with *c*-*Myc*, which is a key proto-oncogene and is enhanced in most human cancers [43]. In this study, ORAI3 down-regulation specifically reduced the expression and activity of c-Myc via the mitogen-activated protein kinase (MAPK) pathway, leading to breast cancer cell arrest in the G_1_ phase. Ay et al. [40] found that high expression of ORAI3 promoted non-small cell lung adenocarcinoma cell proliferation via the phosphoinositide 3-OH kinase (PI3K)/Akt signaling pathway, which was constitutively activated in lung cancer cells and was central to cell proliferation and survival. Schmidt et al. [41] also demonstrated that ORAI overexpression induced the activity of the oncoprotein Akt, which contributed to therapy resistance in ovarian carcinoma cells.

Any structural remodeling and functional changes of ORAI3 may trigger a switch to a more aggressive cell phenotype. Dubois et al. [44] showed that enhanced ORAI3 expression favored heteromerization with ORAI1 to form a novel channel in in vitro models; the remodeled ORAI1–ORAI3 complex might serve as the oncogenic switch in prostate cancer (Fig. 1b). Additionally, the authors found that the relative expression level of the ORAI3 protein in cancer tissues was obviously higher than the level in noncancerous tissues. Overexpressed ORAI3 was shown to encode SOCE in a subset of breast cancer cells that partially substituted for functional ORAI1 channels [45]. Importantly, elevated expression level of the ORAI3 favored the association with ORAI1 to form heteromultimeric, store-independent, arachidonic, acid-regulated channels at the expense of “classical” homomultimeric ORAI1-based SOCE. The “nonclassical” association of ORAI3 and ORAI1 crippled the functions of SOCE, leading to the resistance of malignant cells to apoptosis due to the declining infusion of Ca^2+^. Furthermore, the remodeled ORAI channels promoted cancer cell proliferation via activation of the transcription factor nuclear factor of activated T cells (NFAT), followed by the stimulation of cyclin D1 expression, which is a key rate-limiting controller of the G_1_/S phase transition. Faouzi et al. [39] demonstrated that ORAI3 contributed to the regulation of the cell cycle by the estrogen receptor expressed on breast cancer cells but not normal breast epithelial cells. These authors reported that knockdown of ORAI3 caused a surprising increase in the levels of the well-established tumor suppressors P53 and P21, leading to cell cycle arrest.

STIM and ORAI have also been found to affect the migration of cancer cells. Increasing evidence has shown that tumor migration can be viewed as a Ca^2+^-dependent signaling process, and STIM–ORAI is hijacked by malignant cancer cells to drive the biological functions required for tumor development [46]. In other words, although tumor migration is a complicated and multistep process, STIM–ORAI participates in almost every aspect of tumor cell migration, including the formation of lamellipodia/membrane protrusions at the front edge, cycles of adhesion and detachment, cell body contraction, and tail retraction [47]. Blocking STIM-ORAI with its inhibitor, SKF-96365/2-aminoethoxydiphenyl borate (2-APB), or siRNA-mediated gene knockdown can obviously restrain the migration of hepatocarcinoma [34], breast cancer [37], glioblastoma multiforme [48], pancreatic adenocarcinoma [49], and melanoma cells [50]. The STIM-ORAI-mediated Ca^2+^ influx accelerates focal adhesion (FA) turnover through the constitutively active forms of the small GTPase RAC1 and the Ca^2+^-dependent proline-rich tyrosine kinase 2 (Pyk2) [51]. The efficiency of the assembly and disassembly of FAs decides the speed of cancer cell migration (Fig. 1c). Assembled FAs serve as anchorage points for actomyosin to provide the traction force that moves the cell body forward [52, 53]. At the same time, the STIM-mediated Ca^2+^ signaling enhances contractile forces by regulating the actomyosin reorganization. Actomyosin is a complex of actin filaments and non-muscle myosin II. The actomyosin generates cortical tension with the extracellular matrix or neighboring cells and transmits the contraction to FAs that move the cell body [54]. These findings have corroborated that the STIM1-ORAI-mediated Ca^2+^ signaling exerted comprehensive and crucial functions to promote tumor cell migration by interacting with FA and actomyosin [55]. Moreover, knockdown of ORAI3 reduced the expression levels of cycle D and E1 and finally inhibited the transcriptional activity of NFAT [56]. NFAT is a constitutively active form of the Ca^2+^-dependent transcription factor that plays a critical role in the tissue invasion of tumor cells by promoting the expression of autotaxin and cyclooxygenase 2 (COX2); these factors participate in the epithelial-to-mesenchymal transition [57].

## The burgeoning field of IP_3_R in cancer biology

Growing attention has been paid to the special role of IP_3_Rs in tumorigenesis and tumor metastasis. Over the last 20 years, IP_3_Rs have been regarded as key regulators that control cell death and survival in a variety of cellular systems. Interfering with the amount of IP_3_R-mediated Ca^2+^ transport from the ER to the mitochondria determines the susceptibility of cells to apoptotic stimulation. Because IP_3_Rs can promote senescence and/or apoptosis, the available evidence indicates that down-regulating IP_3_Rs or dampening their activities can decrease cellular sensitivity to apoptotic signaling, finally resulting in the survival of cells with oncogenic features. An in vitro study showed that knockdown of IP_3_R1 prevented apoptosis in bladder cancer cells and rendered them resistant to chemotherapeutics [58]. Conversely, overexpression of IP_3_Rs might increase the sensitivity of cancer cells to cisplatin [59]. As the molecular bridge between the ER and mitochondria, IP_3_Rs are also hijacked by different proto-oncogenes to give rise to cells with oncogenic features [60], such as Akt/protein kinase B (PKB) [61], Bcl-2 family members [62, 63], Bax inhibitor-1 (BI-1) [64, 65], and K-ras-induced actin-interacting protein (KRAP) [66]. Recently, it has become clear that Bcl-2 directly targets the central modulation domain of IP_3_Rs through its tetrahydrobiopterin (BH4) domain to inhibit their functions [61]. The spatiotemporal interaction of BH4 and IP_3_Rs hindered mitochondrial Ca^2+^ accumulation by abrogating Ca^2+^ transport from the ER to the outer mitochondrial membrane (Fig. 1d), therefore, the IP_3_R-BH4 complex counteracted the pressure of pro-apoptotic proteins to protect tumor cells [67]. The Bcl-2 family was also shown to enhance basal Ca^2+^ leakage through sensitization of IP_3_Rs to basal IP_3_ levels lower than the Ca^2+^ concentration in the ER [68, 69]. The low levels of Ca^2+^ in the ER destroyed the mitochondrial Ca^2+^ overload and decreased the susceptibility of the cells to apoptosis. Importantly, a peptide tool that was designed to disrupt the IP_3_R-BH4 complex could effectively induce an intracellular Ca^2+^ overload and provoke cell death in diffuse large B cell lymphoma (DLBCL) cells [70]. However, Kang et al. [71] reported that the invasion and migration of tumor cells were suppressed by caffeine, which is a well-known inhibitor of IP_3_Rs. The expression levels of IP_3_R3 in colon cells were directly related to tumor aggressiveness [72]. These results suggest that the regulatory mechanisms of IP_3_Rs may vary in different types of cancer, and many mechanisms are not fully understood.

## The interaction between STIM/ORAI and IP_3_Rs in cancer biology

The binding of IP_3_ to IP_3_Rs releases intracellular Ca^2+^, leading to a reduction in the Ca^2+^ concentration in the lumen of ER, which in turn activates the STIM sensor to allow extracellular Ca^2+^ to refill the empty ER Ca^2+^ stores across the ORAI in the plasma membrane. In rapidly growing cancers, IP_3_Rs are blocked by a variety of anti-apoptosis proteins, resulting in Ca^2+^ overload in the ER. The harsh microenvironment perturbs the STIM-ORAI functions and induces the accumulation of misfolded proteins in the ER. This triggers an adaptation program referred to as “ER stress.” Chronic ER stress kills normal cells but can contribute to tumor cell dormancy, thereby permitting survival in the stressed environment until more favorable conditions are encountered. Overexpression of STIM could reverse ER stress, implying that Ca^2+^ overload restrains the STIM functions in cancer cells [73]. Oncogenic KRAS mutations could reduce the Ca^2+^ store content in the ER via promoting IP_3_R1 overexpression to suppress agonist-induced Ca^2+^ release and mitochondrial Ca^2+^ accumulation in cancer cells [74]. Nevertheless, the relationships between STIM–ORAI and IP_3_Rs are not completely understood, and further investigations are needed to elucidate the mechanisms by which cancer cells control the functions of STIM-ORAI and IP_3_Rs.

## STIM–ORAI and IP_3_Rs in cancer therapy

Multiple roles of STIM–ORAI and IP_3_Rs in several types of human cancer have made them attractive drug targets for tumor therapy. Inhibiting ORAI1 by pharmacologic antagonists in cultured epithelial cells derived from ESCC patients impeded ESCC cell proliferation, invasion, and migration [75]. Importantly, the growth of ESCC in vivo was significantly suppressed when ORAI1-mediated SOCE was knocked down by siRNA or blocked by pharmacologic inhibitors in xenografted nude mice. SKF-96365 and 2-APB, which are inhibitors of store-operated Ca^2+^ entry, inhibited the growth and metastasis of tumor cells after 1 week of treatment [76, 77]. No increase in metastasis was observed in mouse cancer models [78], even 2 weeks after withdrawal of SKF-96365. Similar phenomena have been found in cervical and esophageal cancer mouse models.

To date, “proof of principle” studies of the Ca^2+^ signal channels have shown that STIM-ORAI and IP_3_Rs either do not differ or are overexpressed in tumor tissues compared to those in normal tissues. However, the roles of STIM-ORAI and IP_3_Rs may be over- or underestimated depending on the use of the pharmacologic inhibitors or siRNA-mediated gene knockdown approaches in cancer cells. Moreover, only a relatively limited amount of information concerning STIM-ORAI and IP_3_Rs is available to date due to their complicated and comprehensive functions in tumor cells. Despite a wealth of data describing their functions, the elucidation of their roles in cancer is still at the beginning stages. How STIM-ORAI and IP_3_Rs affect carcinogenesis in vivo, the relationship between these proteins and Ca^2+^ oscillations in cancer cells, and whether the participation of these proteins in the cancer procedure is a general mechanism need to be investigated.
