# Erratum to: Altered expression of stromal interaction molecule (STIM)-calcium release-activated calcium channel protein (ORAI) and inositol 1,4,5-trisphosphate receptors (IP_3_Rs) in cancer: will they become a new battlefield for oncotherapy?

**DOI:** 10.1186/s40880-016-0116-0

**Published:** 2016-06-14

**Authors:** Jing Wen, Ying-Cheng Huang, Huan-Huan Xiu, Zhi-Ming Shan, Kang-Qing Xu

**Affiliations:** Department of Anesthesiology, The First Affiliated Hospital, Sun Yat-sen University, Guangzhou, 510080 Guangdong P. R. China; Zhongshan School of Medicine, Sun Yat-sen University, Guangzhou, 510080 Guangdong P. R. China

## Erratum to: Chin J Cancer (2016) 35:32 DOI 10.1186/s40880-016-0094-2

After publication of the original article [1], the authors noticed an error in the body of text in the last paragraph of section ‘The structure and function of the STIM and ORAI protein families’. The sentence “The primary intracellular hysiologic functions of STIM and ORAI are straightforward” should read: “The primary intracellular physiological functions of STIM and ORAI are straightforward”.
